# Cortical Activity During an Attack of Ménière's Disease—A Case Report

**DOI:** 10.3389/fneur.2021.669390

**Published:** 2021-07-22

**Authors:** Louise Devantier, Allan K. Hansen, Jens-Jacob Mølby-Henriksen, Michael Pedersen, Per Borghammer, Therese Ovesen, Måns Magnusson

**Affiliations:** ^1^Department of Clinical Medicine, Aarhus University, Aarhus, Denmark; ^2^Department of Oto-Rhino-Laryngology, Regional Hospital West Jutland, Holstebro, Denmark; ^3^Department of Nuclear Medicine, Positron Emission Tomography (PET) Centre, Aarhus University Hospital, Aarhus, Denmark; ^4^Comparative Medicine Lab, Aarhus University, Aarhus, Denmark; ^5^Department of Oto-Rhino-Laryngology, Lund University Hospital, Lund, Sweden

**Keywords:** case report, Menière's disease, neuroimaging, PET, cortical activity

## Abstract

**Background:** Ménière's disease (MD) is a chronic peripheral vestibular disorder with recurrent episodes of vertigo accompanied by fluctuating hearing loss, tinnitus and aural fullness in the affected ear. There are several unanswered fundamental questions regarding MD, one of these being cortical activity during a MD attack. However, it is not possible to plan an investigation in an episodic disease as MD.

**Objective:** To visualize cortical activity during an attack of MD.

**Method:** ^18^F-FDG PET scans were used to visualize cortical activity in a 62 years old male suffering from definite MD. Two ^18^F-FDG PET scans were performed. One to show activity during the attack and one to show normal baseline brain activity 7 days after the attack.

**Results:** A number of low-magnitude fluctuations in the ^18^F-FDG FDG uptake were found in ^18^F-FDG PET examination following the MD attack compared to the patient's own baseline ^18^F-FDG FDG scan. Across both hemispheres no significant changes were seen. However, reduced activity was observed in most of the orbitofrontal, frontal cortices as well as Heschl's gyrus and insula.

**Conclusion:** This is the first neuroimaging showing alteration of brain activity during an attack in a patient with MD. No strong focal alterations was seen. It is noteworthy that the decreased activity observed was in the insula and Heschl's gyrus that seems to be core areas for processing information from the labyrinth. It is also of interest that decreased activity rather than hyperactivity was observed.

## Introduction

Ménière's disease (MD) is a chronic peripheral vestibular disorder associated with an accumulation of endolymphatic fluid in the inner ear, called endolymphatic hydrops (1). Patients suffer from recurrent episodes of vertigo accompanied by fluctuating hearing loss, tinnitus and aural fullness in the affected ear. There are several unanswered fundamental questions regarding MD including that the endolymphatic hydrops cannot account for all the symptoms patients with MD experience (2).

In general the knowledge of vestibular cortical processing in humans is variegated and inhomogeneous (3) and the alterations of cortical activity and processing MD patients endure during an attack are non-existing. We have previously visualized baseline brain activity and brain activity after natural vestibular stimulation using ^18^F-FDG PET in healthy participants (4). During a later project we included MD patients in order to compare their brain activity during baseline and vestibular stimulation to the group of healthy participants (5). One of these MD patients suffered from a MD attack immediately prior to the PET examination and thus enabled us an unique opportunity to visualize cortical brain activity during the attack.

## Method

### Patient

The patient was a right-handed 62 years old male suffering from definite MD for more than 2 years on the right ear. The diagnostic criteria for definite MD is defined by the Bárány Society (6) as (1) Two or more spontaneous episodes of vertigo, each lasting 20 min to 12 h. (2) Audiometrically documented low- to medium-frequency sensorineural hearing loss in one ear, defining the affected ear on at least one occasion before, during or after one of the episodes of vertigo. (3) Fluctuating aural symptoms (hearing, tinnitus or fullness) in the affected ear. (4) Not better accounted for by another vestibular diagnosis. The patient had a sensorineural hearing loss of 70–80 dB across all frequencies on the right ear. On the left ear, he had presbycusis with normal hearing in the lower frequencies. His MD treatment consisted of a grommet in the right tympanic membrane and daily Betahistine (dosage: 8 mg × 3). The patient was a non-smoker, and had no other medical disorders including no psychiatric disorder. Vestibular testing 3 weeks prior to the MD attack showed a normal video Head Impulse Test (vHIT) for all three semicircular canals on both sides (EyeSeeCam, Interacoustics, Denmark). Bone-conducted Ocular Vestibular Evoked Myogenic Potential (oVEMP) (Eclipse, Interacoustics, Denmark) showed no response on either sides. Air-conducted cervical Vestibular Evoked Myogenic Potential (cVEMP) (Eclipse, Interacoustics, Denmark) showed normal response on the left side, but no response on the right side, where the grommet was placed. No spontaneous nystagmus was observed during videonystagmography 1 h prior to any of the ^18^F-FDG PET scans (VisualEyes, Interacoustics, Denmark). Head shake test 1 h prior to the attack was positive toward the right (affected) ear, (VisualEyes, Interacoustics, Denmark). The head shake test was normal on two other visits, 1 week prior and 1 week after. The patient described this particular attack comparable to those he had previously perceived. However, he considered the current attack as a small attack. He initially experienced tinnitus and aural fullness in the right ear, followed shortly after by rotatory vertigo and nausea. The attack lasted 20–25 min. The patient did not experience any attacks 3 weeks prior to the attack or in the following week before the baseline ^18^F-FDG PET examination.

Informed oral and written consent to participate and publish the presented data and images were obtained from the patient. The study was approved by the Central Region of Denmark Research Ethics Committee (no: 1-10-72-135-16).

### ^18^F-FDG-PET Procedures

The patient was seated comfortably and strapped into a chair. The chair has a built-in headguard in order to minimize head movements whilst seated in the chair. The patient wore noise-canceling in-ear headphones (QuietControl 30 wireless headphones, Bose, USA) to minimize auditory stimulation and sleep-goggles to avoid visual stimulation.

Whilst seated the patient received a bolus injection of radioactive ^18^F-FDG [170 Mq (±10%)]. No vestibular stimulation was applied as the ^18^F-FDG PET examination scan was scheduled as a baseline ^18^F-FDG PET scan. The MD attack initiated ~5 min after injection of the ^18^F-FDG bolus and lasted 20–25 min. Subsequently, the patient was transferred to the HRRT PET scanner, and a 66 min PET imaging session was performed. Almost the entire injected dose of ^18^F-FDG is cleared from the blood stream during the first 40 min (7). During this time span, the ^18^F-FDG tracer is irreversibly trapped due to phosphorylation in neurons proportionally with their metabolic need (8). The subsequently PET scan reveals the reginal cerebral uptake of ^18^F-FDG during the 30–40 min after the ^18^F-FDG bolus injection as the half-life of the radioactive isotope ^18^F-FDG is approximately 110 min (9).

A baseline ^18^F-FDG PET scan was obtained seven days later for comparison with the brain activity during this attack. After the ^18^F-FDG bolus was injected the patient remained seated for 35 min before he was transferred to the PET scanner. The ^18^F-FDG PET scans were on both occasions initiated precisely 60 min post ^18^F-FDG bolus injection.

Complete details of the PET scanner have been published previously (10).

## Results

### Brain Activity During Ménières Attack

**Figure 1:**
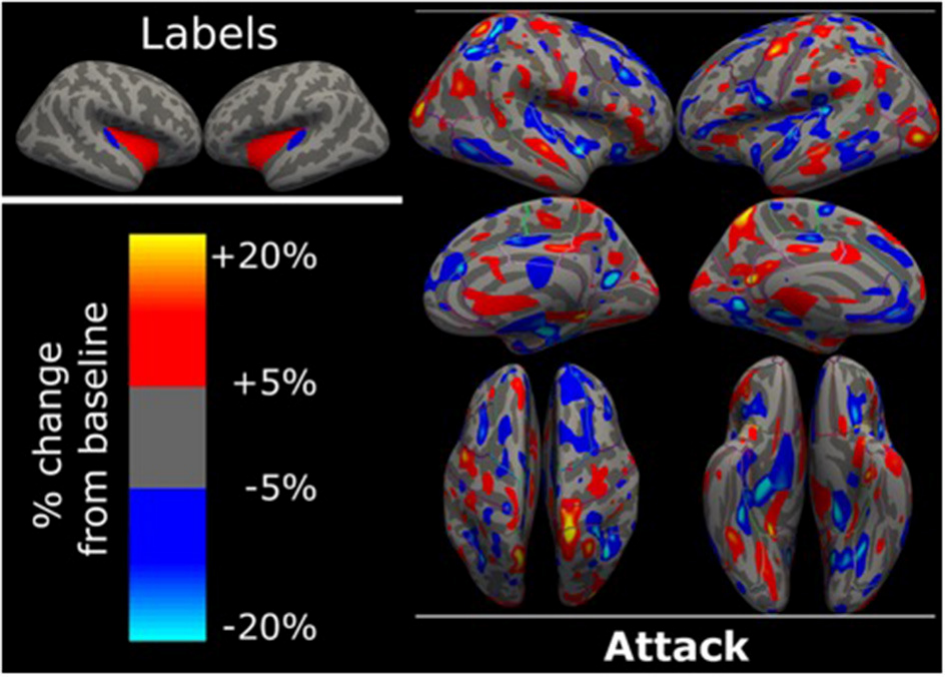
Surface based analysis. Surface based analysis in FreeSurfer showing percentage changes of ^18^F-FDG uptake during MD attack compared to the patient's own baseline ^18^F-FDG scan. There were no significant changes of more than 20% change between the ^18^F-FDG uptake during MD attack compared to the patient's own baseline scan obtain 7 days later. Labels for anatomical orientation are shown in the upper left corner. Blue is Heschl's gyrus, red is insula, as defined by the Desikan-Killeany atlas. Dark gray is sulci, light gray is gyri.

The ^18^F-FDG PET examination following the MD attack revealed no significant changes across both hemispheres compared to the patient's own baseline scan (Figure 1). Only a number of low-magnitude variations (±20% changes) in the FDG uptake were found between the two ^18^F-FDG PET examinations. These changes might represent random fluctuations. However, reduced activity was observed in most of the orbitofrontal, frontal cortices as well as Heschl's gyrus and insula.

## Discussion

To our knowledge, these are the first images of the brain activity during a vertigo attack in a MD patient. The attack did not elicit strong focal alterations in brain activity, in contrast to what is generally observed during, for instance, epileptic seizures, where an increased glucose uptake of 50–100% is common (11). This lack of enhanced activity is likely explained by the fact that MD is a peripheral vestibular disorder of the inner ear and not a central neurological disease. However, we speculate that the pathophysiological response on the MD attack instigates an immediate central, possible cerebellar inhibition.

To avoid visual and auditory stimulation all participants in the originally planned study wore sleep-goggles and noise-canceling in-ear headphones until the injected dose of ^18^F-FDG was cleared from the blood stream. The MD attack began shortly after the ^18^F-FDG injection and thus bestowed a unique opportunity to visualize cortical activity during an MD attack.

The patient wore the sleep-goggles during the entire attack and consequently there is no information on the presumed nystagmus during the attack. However, the observation of headshake nystagmus directed toward the affected ear, directly prior to the attack corroborate the idea of an “irritative” state in an active MD (12–14). The patient described tinnitus, aural fullness, vertigo and nausea during the attack, but not the pathogenetic complaint of hearing loss. The patient's hearing on the right (sick) ear had deteriorated with previous MD attacks resulting in a 60–70 dB hearing loss. This and the fact that he wore noise-canceling ear-phones in a silent room, explains the lack of perceived hearing loss during the attack. It is unclear if the decrease in ^18^F-FDG uptake in the central auditory area (Heschl's gyrus) can be explained by alterations in the cochlear function during the attack.

The posterior part of insula is believed to be a core area for processing vestibular stimuli (15) and the adjoining medial part of Heschl's gyrus have been suggested to be a possible primary vestibular cortex (4, 5). If that is the case, Heschl's gyrus is a primary cortical area for the entire labyrinth and not merely the cochlear part (4, 5). Baseline ^18^F-FDG uptake in MD patients have been shown to solely differ with lower cortical activity in the medial part of Heschl's gyrus compared to healthy controls (5). It is noteworthy that there is decreased activity in these areas during the attack compared to the patient's own baseline brain activity. Previous studies have also shown increased activity in these areas during artificial vestibular stimulation as caloric, sound or galvanic stimulation (16–18).

The predominance of reduced activity in most of the orbitofrontal and frontal cortices is also interesting. These areas are considered of importance for higher cerebral functions such as planning and social interaction, supporting the observations and recognized loss of social interest and drowsiness described in motion sickness, which is another form of mainly vestibular-induced nausea. Our finding further corroborates with the decreased intellectual capacity often described by patients during situations of vestibular induced nausea.

## Founding

This project has received founding from The Toyota Foundation, The Oticon Foundation, Hans Skouby's Foundation, Aase and Ejnar Danielsen's Foundation, Knud and Edith Eriksen's Memorial Foundation, The Ménière and tinnitus Foundation, The Augustinus Foundation, The A.P. Møller Foundation for the Advancement of Medical Science, Health Research Fund of Central Denmark Region.

## Conclusion

This is the first neuroimaging showing alteration of brain activity during an attack in a patient's with MD. No strong focal alterations were seen. However, it is noteworthy that decreased activity was seen in the insula and Heschl's gyrus that can be perceived as core areas for processing information from the labyrinth.

## Data Availability Statement

The original contributions presented in the study are included in the article/supplementary material, further inquiries can be directed to the corresponding author/s.

## Ethics Statement

Written informed consent was obtained from the individual for the publication of any potentially identifiable images or data included in this article.

## Author Contributions

LD collected the data. LD, PB, and AH performed the statistical analysis. LD and MM wrote the first draft of the manuscript. All authors contributed to conception, design of the study, manuscript revision, read, and approved the submitted version.

## Conflict of Interest

The authors declare that the research was conducted in the absence of any commercial or financial relationships that could be construed as a potential conflict of interest.
